# Task performance errors and rewards affect voluntary task choices

**DOI:** 10.1007/s00426-023-01908-7

**Published:** 2024-01-04

**Authors:** Markus Wolfgang Hermann Spitzer, Sebastian Musslick, Janina Janz, Andrea Kiesel, David Dignath

**Affiliations:** 1 Department of Psychology, Martin-Luther University Halle-Wittenberg, Halle, Germany; 2 Institute of Cognitive Science, Osnabrück University, Osnabrück, Germany; 3 Carney Institute for Brain Science, Brown University, Providence, RI 02906 USA; 4 Albert-Ludwigs-Universität Freiburg, 79085 Freiburg, Germany; 5 Eberhard Karls University of Tübingen, 72076 Tübingen, Germany

## Abstract

Humans are remarkably flexible in adapting their behavior to current demands. It has been suggested that the decision which of multiple tasks to perform is based on a variety of factors pertaining to the rewards associated with each task as well as task performance (e.g., error rates associated with each task and/or error commission on the previous trial). However, further empirical investigation is needed to examine whether task performance still influences task choices if task choices are rewarded but task performance is not. Accordingly, we exposed participants to a novel reward-varying voluntary task switching paradigm where the reward for the performed task gradually decreased while the reward associated for the alternative task was unchanged. Importantly, we rewarded participants’ task choices before participants performed the task to investigate the effect of rewards independent from task performance. We examined the effect of (i) reward, (ii) error rates associated with each of the two tasks, and (iii) error commission in the previous trial on voluntary task choices. As expected, we found that participants’ task selection was influenced by reward differences between task choices. In addition, error rates associated with a task also influenced task selection, with participants requiring larger reward differences to switch to a task associated with relatively higher error rates, compared to switching to a task with relatively lower error rates. However, errors in *n *− 1 did not influence participants’ probability to switch to the alternative task. These findings contribute to an ongoing discussion on the influence of task performance on task selection.

**Supplementary Information:**

The online version contains supplementary material available at 10.1007/s00426-023-01908-7.

## Introduction

Humans show remarkable flexibility in switching from one task to another. While the past decades of psychological research primarily focused on the cognitive mechanisms that enable flexible task switching (for reviews see: Kiesel et al., 2010; Koch et al., 2018; Vandierendonck et al., 2010), recent work began to investigate how factors which influence task performance, such as rewards, error rates associated with a task, and errors in *n *− 1 influence people’s decision about when to disengage from their current task to perform another task (Braun & Arrington, 2018; Fröber & Dreisbach, 2016, 2020; Fröber et al., 2019; Jurczyk et al., 2019; Spitzer et al., 2022). Elucidating these factors underlying task choice may help us better understand human decision-making in daily life, including voluntary decisions of switching from one task to another.

So far, voluntary task switching studies provided evidence that task performance, as well as performance-contingent rewards, can influence participants’ decision of switching from one task to another. For instance, prior work has demonstrated that reductions in *task performance* can incentivize participants to disengage from the currently performed task (Dignath et al., 2015; Gilzenrat et al., 2010; Mittelstädt et al., 2017, 2018, 2019; Monno et al., 2021; Schuch & Dignath, 2021; Spitzer et al., 2022). Other research demonstrated that reductions in *rewards* can drive participants to disengage from a task (Braun & Arrington, 2018). Critically, rewards provided in the study by Braun and Arrington (2018) were contingent on participants’ task performance (with rewards provided only for accurate responses). Thus, it remains an open question whether humans would still consider their task performance if task choices are purely driven by monetary incentives but not influenced by task performance, i.e., participants receive rewards independently from their accuracy. Here, we sought to address this question by exposing participants to a task environment in which they received rewards for their task selection, irrespective of their task performance. We then asked whether factors associated with task performance—such as error rates associated with a task and/or errors in *n *− 1—would still influence participants’ decision to switch to an alternative task even though they did not affect whether participants’ gained rewards.

Theories of control allocation suggest that task selection is guided by weighing the benefits of performing a task against its respective cognitive control costs associated with task performance (Lieder et al., 2018; Musslick et al., 2015; Shenhav et al., 2013; Silvestrini et al., 2022). According to the expected value of control (EVC) theory, several factors determine the decision to switch from one task to another task such as monetary rewards and costs associated with the amount of control associated with performing a task, error commission, reconfiguration costs associated with adjusting cognitive control (Musslick et al., 2015; Shenhav et al., 2013). While previous instances of the EVC theory accounted for the effects of individual factors on task choice and performance (Grahek et al., 2020; Musslick et al., 2018, 2019b), it remains unclear *whether* and, if so, *to which degree* these factors (i.e., reward differences between tasks,^1^ error rates associated with a task, and errors in *n *− 1) affect voluntary task choices. More specifically, empirical investigation is needed to probe whether these factors affect voluntary task choices in an additive (as main effects) and/or multiplicative fashion (interactions plus main effects). For instance, do very small reward differences (e.g., 1 cent) influence participants’ decision to disengage from a task associated with small cognitive control costs due to relatively low task performance demands to switch to another task associated with high cognitive control costs due to relatively high task performance demands? Or are cognitive control costs (also) considered when reward differences between tasks are very large? Critically, previous empirical studies on effort-based decision-making mostly considered a similar question, but either exposed participants to *only one* task with varying task difficulty, asking participants to select between difficulties within the task (Chong et al., 2017; Gilzenrat et al., 2010; Westbrook et al., 2013, 2020), or varying task difficulty *between* tasks but providing participants with random rewards for their task choice and only for correct responses (Dreisbach & Jurczyk, 2022; Jurczyk et al., 2019). Here, we seek to extend this work to scenarios where participants had to select *between* two tasks on a trial-by-trial basis where rewards were contingent on task selection but non-contingent on task performance. Moreover, the present study seeks to examine the possible additive or multiplicative nature of the influence of the three factors—reward differences, error rates associated with a task, and errors in *n*−1—within one experimental setup. In the following sections, we review prior evidence suggesting effects of (a) reward difference, and (b) error rates associated with a task and errors in n-1on choice behavior in voluntary task switching.

## Influence of monetary rewards on voluntary task switching

The effect of incentives on task choice has recently been studied in voluntary task switching (Braem, 2017; Braun & Arrington, 2018; Dreisbach & Jurczyk, 2022; Fröber & Dreisbach, 2016; Fröber et al., 2018, 2019; Jurczyk et al., 2019). For instance, Braun and Arrington (2018) found that people are more likely to switch to an alternative task if this alternative task was associated with a higher reward than the currently performed task. In a voluntary task switching paradigm, varying rewards for two possible tasks (i.e., identifying the color or shape of a stimulus) were presented before the presentation of the stimulus. Notably, the rewards for each task changed as a function of task choice: the reward for the performed task decreased with a probability of 50% while the reward for the alternative task increased with a probability of 50%. Results showed that the probability of switching to an alternative task was proportional to reward differences in favor of the alternative task (for further studies on reward-dependent switch rates see^2^: Braem, 2017; Fröber et al., 2018, 2019; Fröber & Dreisbach, 2016, 2020; Jurczyk et al., 2019). Furthermore, the study by Braun and Arrington (2018) revealed that participants refrained from task switching if the rewards of the alternative task were similar to the rewards provided for the performed task. This suggests that not the total reward associated with each task, but rather the relative reward *difference* motivated participants to switch to an alternative task. This comports with earlier findings suggesting that participants avoid the costs of switching between tasks (Arrington & Logan, 2004, 2005; Arrington & Reiman, 2015; Kessler et al., 2009; Mayr & Bell, 2006; Yeung, 2010), supporting rational accounts of control allocation which propose that internal cost/benefit signals (e.g., due to a task-reconfiguration during task-switching) are integrated with external costs/benefit signal (e.g., rewards) into a joint utility function (Musslick et al., 2015, 2019b).

## Influence of error rates and errors in n-1 on voluntary task switching

There is mounting evidence suggesting that overall task performance associated with a specific task influences participants’ task choices, suggesting that participants choose tasks associated with overall lower error rates over tasks associated with higher error rates (Kool et al., 2010; Shenhav et al., 2017; Westbrook et al., 2013; Wisniewski et al., 2015). In addition, recent empirical work suggests that error rates associated with a task as well as errors in *n *− 1 affect task choices (Spitzer et al., 2022). In a set of three experiments, Spitzer et al. (2022) exposed participants to a novel voluntary task switching paradigm without any instructions on how often to switch tasks or how often to select each task (see Arrington & Logan, 2004 for an example of the instructions used by many voluntary task switching studies) but rather motivated voluntary task switches by dynamic changes in the task environment. Results showed that error rates associated with the performed task, error rates associated with the alternative task, and errors in *n *− 1 affected participants’ voluntary choice to switch to the alternative task with the highest switch probabilities if the performed task was associated with high error rates, the alternative task was associated with low error rates, and after errors in *n *− 1. These results corroborated modeling predictions that error rates as well as errors in n-1 influence voluntary task choices (Musslick et al., 2015).

To summarize, previous work supported the assumption that both rewards and task performance (e.g., error rates and errors in *n *− 1) modulate voluntary task choices (Braun & Arrington, 2018; Spitzer et al., 2022). Yet, it is still unclear whether task performance affect voluntary task choices when rewards purely depend on task choices and not task performance—and if they do, how they would interact with receiving incentives. Thus, this research project aims to test how these factors (i.e., reward differences, error rates, and errors in *n *− 1) contribute to a joint utility function guiding decision making.

## The present research

In the present study, we sought to investigate the effect of reward difference, task (associated with different error rates), and errors in n-1 on voluntary task choices. We examined the main effects and interactions between these factors in a voluntary task switching paradigm using a double registration procedure. In this paradigm, participants first voluntarily selected (*task selection*, first registration of a response) one of two tasks—a color discrimination task or a motion discrimination task—and subsequently responded to the presented stimulus (*task performance*, second registration of a response). The two tasks were selected as they allow varying the signal-to-noise ratio of the task (and with that the error probability of the task), without varying the task identity (Musslick et al., 2019a; Ritz & Shenhav, 2019; Shenhav et al., 2018; Spitzer et al., 2019, 2022). Moreover, rewards gained for the selected task dynamically decreased while the rewards potentially gained for the alternative task remained on the same level. Participants received task-dependent monetary rewards right after the task selection (first registration), i.e., before the task performance (second registration), and thus, rewards gained for a selected task were independent of task execution. Furthermore, in contrast to previous studies on voluntary task switching (Arrington & Logan, 2004, 2005; Brüning & Manzey, 2018; Brüning et al., 2020; Dignath et al., 2015; Mayr & Bell, 2006; Poljac & Yeung, 2014; Yeung, 2010), participants were not instructed about specific strategies how to select tasks (e.g., a mental coin flip, see Arrington & Logan, 2004), or how often to select each task (e.g., Brüning & Manzey, 2018; Brüning et al., 2020; Mittelstädt et al., 2018, 2019; Monno et al., 2021).

Building on the behavioral and simulation studies reviewed above, we expected that participants gradually are more likely to switch to an alternative task as this task becomes relatively more rewarding. As the commission of an error indicating an increase in cognitive costs associated with a task, we also expected higher switch rates after errors, compared to accurate responses (see Spitzer et al., 2022, Exp. 2). While Experiment 1 did not control for task differences, Experiment 2 and Experiment 3 directly manipulated the signal-to-noise ratios differently between the two tasks, rendering one task consistently harder than the other. For both experiments, we expected again a main effect for reward difference and errors in n-1. Further, based on the observation that participants avoid more difficult tasks (i.e., tasks associated with high error rates; see Spitzer et al., 2022), we expected a main effect of task (due to the manipulated the signal-to-noise ratios which should increase task differences), with a high probability to switch away from the relatively more difficult task. We formulated no a priori hypotheses regarding a possible interaction between different factors for all three experiments.

So far, it is unclear whether the two effects of rewards and task performance are integrated in an additive, or multiplicative (interactive) fashion, to determine task choice. Previous work suggests that such an integration might occur based on a common currency, such as negative affect (e.g., Delgado, 2007; Dignath et al., 2019). This comports with theoretical and computational work (Musslick et al., 2015; Shenhav et al., 2013; Silvestrini et al., 2022; Silvetti et al., 2018), as well as empirical work applying one task (Chong et al., 2017; Gilzenrat et al., 2010; Westbrook et al., 2013, 2020) emphasizing that both rewards and task performance are consolidated into a common expected value of choosing a particular task. While thus far, computational models considered how both rewards and cognitive costs associated with each task are considered in voluntary task choices (Musslick et al., 2015; Shenhav et al., 2013; Silvestrini et al., 2022; Silvetti et al., 2018), empirical evidence is missing to show that rewards and cognitive costs associated with task performance (such as error rates and errors) are also considered when deciding which of two tasks to select (for evidence on task difficulty affecting within task choices see: Chong et al., 2017; Gilzenrat et al., 2010; Westbrook et al., 2013, 2020). Moreover, it remains to be investigated how errors independently from rewards affect voluntary task choices. To inform further model and theoretical development concerning the interplay of rewards and cognitive control costs, we conducted three empirical experiments.

## Experiment 1

In the first experiment, we sought to establish that increasing reward differences^3^ favoring the alternative task would result in voluntary task switches towards the task associated with relatively higher rewards. We additionally expected higher switch rates after errors, compared to accurate responses, as errors signal an increase in cognitive control costs associated with the performed task (Musslick et al., 2015; Silvestrini et al., 2022).

## Methods

### Transparency and openness

The raw data and analysis scripts of all experiments are available via the Open Science Framework at https://osf.io/epx8b/. The present study was not preregistered. The data was collected in December 2019. This experiment considered a target population of 18–45-year-old right-handed males and females from Germany.

### Participants

Forty right-handed participants (32 females; mean age = 22.7; SD = 2.7) with normal or corrected to normal vision participated in this experiment in the lab. Before the start of the experiment, participants signed a consent form. The reimbursement of the experiment depended on participants’ task choice and ranged between 6 and 8.5 Euros.

An a priori power analysis was calculated with the simr package (Green & Macleod, 2016) to examine the minimal expected effect size for reward difference which was our main effect of interest. This power analysis was based on 100 simulations and suggested a power of 85% with a 95%-confidence interval (95%-CI) between 82 and 88% for a minimal effect for reward difference with an effect size (beta) of 0.015 and an alpha level of 0.05. Another power analysis on the second half of the data for a minimal effect for reward difference and based on 100 simulations suggested a power of 92% with a 95%-confidence interval (95%-CI) between 87 and 95% with an effect size (beta) of 0.30 and an alpha level of 0.05.

### Stimulus

Stimulus presentation and recording were controlled with the jsPsych software (de Leeuw, 2015) applied on a Fujitsu EPrimo P920 computer and a 24-in. screen with a refresh rate of 144 Hz and a resolution of 1920 × 1080. Stimuli were a random-dot motion kinematogram implemented with the rdk-plugin (Rajananda et al., 2018; but also see: Strittmatter et al., 2022). The motion task consisted of 200 black randomly moving dots on a grey background with 40% of these dots moving coherently in an upward or downward direction while the remaining dots moved randomly. The color task consisted of blue and red dots moving in a random direction on a grey background but with 65% of the dots colored in one color (blue or red) and the other 35% of the dots colored in the other color. The signal-to-noise ratios were chosen based on previous experiments so that an overall error rate of 10% was expected (Spitzer et al., 2019, 2022).

### Procedure

**Fig. 1:**
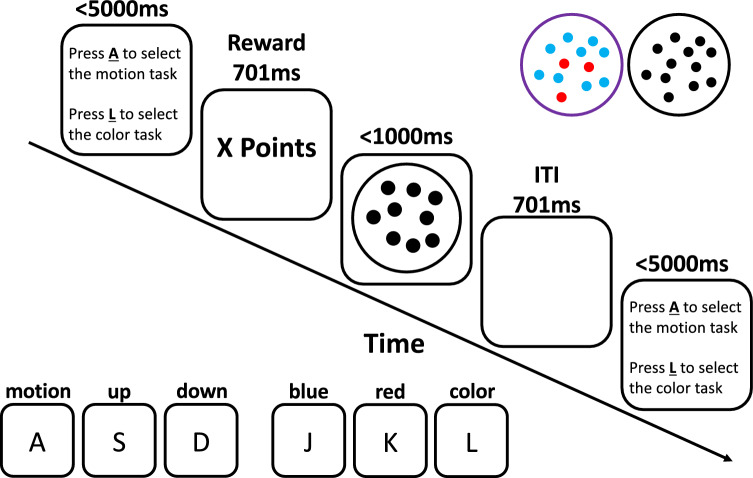
Example trial sequence of the voluntary task switching paradigm with double registration. In the first registration of response, participants selected the motion or the color task. After the task selection, participants received a reward ranging between 40 and 100 points (see text and Fig. 2 for the reward schedule). In the second registration of response, participants responded to the motion or color task by indicating whether most of the dots move upwards or downwards (motion task), or whether most of the dots were colored in blue or red (color task). The stimulus presentation was followed by a blank screen with an ITI of 701 ms

The sequence of events in a trial is depicted in Fig. 1. A trial begins with a task selection phase prompting participants to select one of the two tasks. Participants could select the motion task by pressing the “A” key (left ring finger) and the color task by pressing the “L” key (right ring finger) on a QWERTZ keyboard. If participants did not select one of the two tasks within 5000 ms, the task performed on the previous trial was automatically selected. If no task was selected at the very first trial, the task selection automatically chose a random task. These trials were excluded before the data analysis. After the task selection phase, participants received the reward associated with the chosen task. Depending on the task selection, a color or motion random-dot kinematogram stimulus was presented next and participants had to respond to the stimulus within 1000 ms. For the motion task, participants were instructed to respond by pressing the “S” key (left middle finger) if most of the dots were moving upward and the “D” key (left index finger) if most of the dots were moving downward. In the color task, participants had to press the “J” key (right index finger) for mostly blue dots and the “K” key (right middle finger) for mostly red dots. Responses before the presentation of the stimulus were not recorded. Performance-related feedback was not provided.

**Fig. 2:**
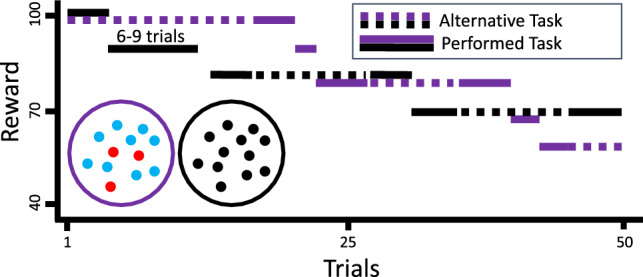
A task choice sequence example. Black lines denote the motion task and purple lines denote the color task. Line types denote whether participants selected and performed the task. At the beginning of each block (trial: 1; reward: 100), participants received 100 points for both tasks, irrespective of which task they selected (the motion task is selected first in this example sequence). Rewards decreased for the selected task with increasing repetitions on this task (indicated as black solid line; note the drop of the black solid line after several trial repetitions). The reward for the alternative, non-selected task did not decrease (indicated as purple dashed line) until participants decided to switch tasks. Participants received points as rewards and were told task 100 points converted to 1 cent. Points were replenished after each block. Note that rewards were contingent on task selection but non-contingent on task performance

Participants were able to accumulate rewards in the form of points. For every 100 points, participants received 1cent. At the beginning of each block, participants could gain 100 points for selecting either task. The number of points participants were able to gain changed throughout each experimental block in the following manner: after 6–9 times of selecting the same task, the points gained for this task decreased by 10 points. Rewards did not decrease on each trial to rule out any expectations about when rewards would potentially drop. The minimum point level participants could reach was 40 points as each block consisted of 50 trials, respectively. The main experiment consisted of 17 blocks. The number of points participants could potentially gain for the non-selected task remained the same. With these settings, points remained at the same level or decreased in ongoing trials within a block and replenished to baseline levels after the completion of each block (see Fig. 2 for an exemplary sequence of rewards). Participants were told that the points of the selected task, but not the non-selected task, decreased over trials. Participants were instructed to gain as many points as possible and that they would receive 1 cent for every 100 points gained during the experiment summing up to a maximum total of 6.5 Euros for their participation. At the same time, participants were instructed to respond as accurately and fast as possible on each trial. The explicit instructions for participants were: *“You will now start the experiment. Before each trial, you can select the task which you would like to do. Remember that you will gain points for each task selection. For every 100 points, you will earn 1 cent. After some trials, the number of points is reduced for the task you select. The points for the other task are not reduced. Please be as fast and accurate as possible on the task response.”.* Before the start of the experiment, participants were able to practice how to respond to each task for ten practice trials in which—unlike in the main experiment—they were provided with response contingent feedback. Participants were able to take self-paced breaks between the blocks.

### Independent and dependent variables

The main analysis included the following three independent variables. The difference in rewards gained for the two tasks (*reward difference*), is computed as the reward of the alternative task minus the reward of the performed task. Positive values of reward difference indicate that the alternative task was associated with a higher reward than the performed task. In addition to reward differences, two categorical variables were considered: *task*, indicating which of the two tasks (the color task or the motion task) participants performed on the previous trial, and *error*_*n-1*_ indicating whether the previous response was correct or incorrect. The probability to switch to the alternative task served as the dependent variable of the main analysis.

### Data analysis

The statistical analysis was carried out with the R software (R Core Team, 2013). Logistic mixed models were conducted with the lmer package (Bates et al., 2014) for all dependent variables with binary outcomes (correct/incorrect or switch/stay). Linear mixed models were conducted with the lmerTest package (Kuznetsova et al., 2017) for all RT analyses. Linear and logistic mixed models were considered as they are less Type 1 error-prone compared to analysis of variance (Judd et al., 2017; Quené & Van Den Bergh, 2004, 2008). Plots were generated with the sjPlot package (Lüdecke, 2020).

The result section first reports average task choices, as well as the performance of each task in terms of task choice latencies (i.e., RTs for task selection), RTs for task performance, and error rates on repetition trials. Note that switch trials were excluded from these analyses as switching tasks was reported to increase RT and error rates (Kiesel et al., 2010; Vandierendonck et al., 2010).

The result section then reports the main analysis. The main analysis comprised the three independent variables reward difference, error_n-1_, and task. All interactions were included as fixed effects to investigate their potential effect on voluntary task switches. A random intercept for each participant was included to account for individual differences between participants’ overall switch rates. In addition, the random slope variables were selected with the following procedure. First, the most complex random effect structure including all random slope variables (reward difference, error_n-1_, and task) was selected and then variables were successively dropped until the random effect variables revealed no singularity (Bates et al., 2015). If more than one model revealed no singularity but an equal complex model structure (e.g., two out of the three variables were included), then the goodness of models with no singularity was computed with the Bayesian information criterion (BIC) (Burnham & Anderson, 2002). Based on this procedure, the two random slope variables reward difference and task were selected as main effects in the model formula. Note that after this procedure, the fixed effect results of potential other random effect structures were also examined which replicated the result pattern of the selected random effect structure.

As outlined above, voluntary task switches towards the alternative task were expected if this task was associated with gaining higher rewards compared to the performed task, reflected with a positive regression slope. In addition, increased switch rates towards the alternative task were expected after errors compared to accurate responses, as would be reflected with a positive regression slope for error_n-1_. As error rates were expected to be similar across both tasks, the task factor was expected to have no significant effect on participants’ choice behavior. In addition, all interactions between these three factors were explored, but without prior hypotheses on the interaction effects.

## Results

**Fig. 3:**
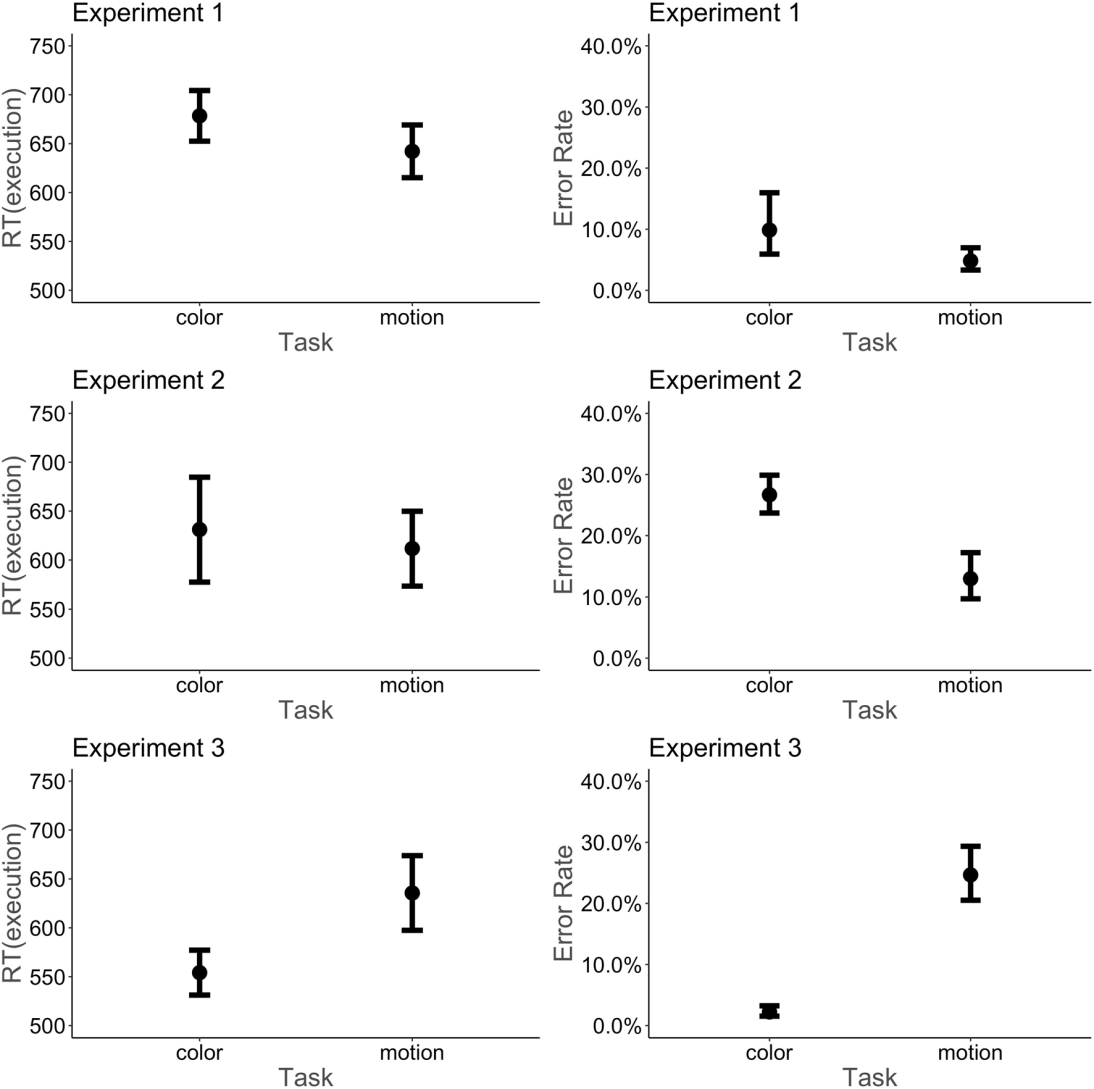
Differences in tasks across experiments. Each row illustrates the RT in milliseconds (left panel) and error rates (right panel) for the color and motion task, respectively, across all three experiments. Reaction times were faster and error rates lower on the motion task than on the color task in Experiments 1–2. However, the effect of task increased with respect to error rates from Experiment 1 to Experiment 2. This result pattern reversed in Experiment 3, with faster RT and lower error rates associated with the color task compared with the motion task

**Fig. 4:**
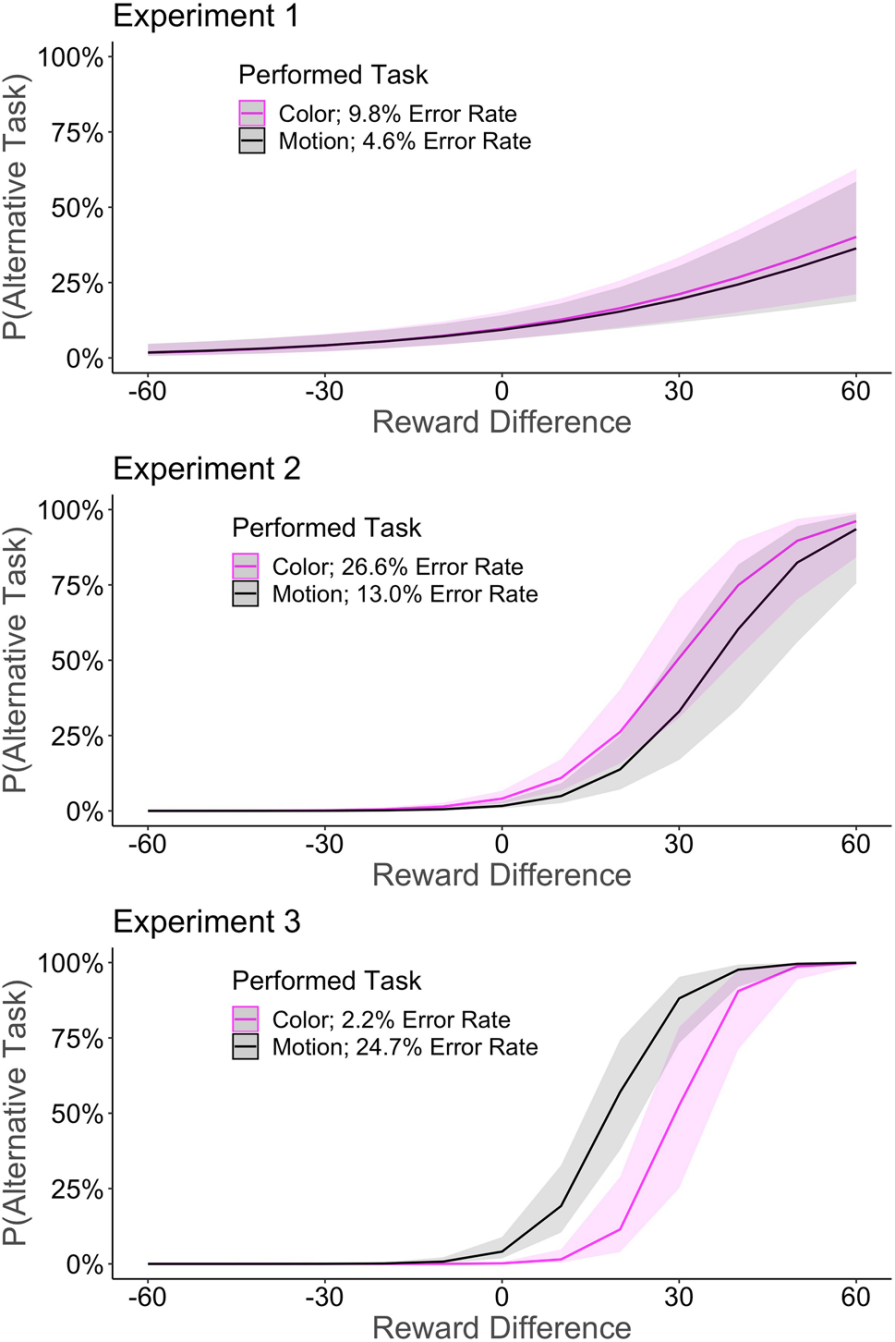
The probability to switch to the alternative task as a function of reward difference and task. Each row illustrates the result of one experiment, respectively. Solid lines indicate the fits of the logistic mixed model, shades indicate the estimated standard error of the mean. The probability to switch to the alternative task increases with increasing differences in rewards. However, switches away from the task with lower error rates (Exp 2–3) were more likely to occur for higher reward differences compared with switches away from the task with higher error rates. More positive reward differences indicate higher payoffs on the alternative task

The effects of task on participants’ RTs and error rates are plotted in Fig. 3. The results of the main analysis are depicted in Fig. 4. Before the statistical data analyses, data sets of two participants with accuracies below 55% were excluded. This cut-off point was set before the data analysis. In addition, one participant who never switched tasks was excluded as this participant may have not understood task instructions to maximize rewards. In addition, the first trial of each block (2% of the trials), all responses below 200 ms (4.4% of all trials) and all trials without a response (4.4% of all trials) or a task selection (0.7% of all trials) were excluded. The average switch rate was 16.3% (SD = 18.5).

### Average task choices, task choice latencies, RTs, and error rates

The motion task was selected with an average of 51.84% (SD = 7.81) of the trials. The main effect of task on task choice latencies was not significant (*b* = 3.17; *t* = 0.87; *p* = 0.390). Participants RTs were 36 ms faster and they made ≈5% less errors on the motion task compared to the color task (RT: *b* = *−* 18.16; *t* = *−* 3.28; *p* = 0.002; Error rate: *b* = *−* 0.41; *z = −* 2.65; *p* = 0.008), with an average response time of 645 ms and 4.6% errors for the motion task and 681 ms and 9.8% errors for the color task. We additionally report an analysis on how individual differences between participants in RTs and error rates affected their task choice in S1.

### Main analysis

The probability to switch to the alternative task as a function of reward difference, error_n-1_, and task*.* In line with expectations, the main effect of reward difference was significant (*b* = 0.02; *z = *4.21; *p* < 0.001), with an increasing probability to switch to the alternative task with higher difference in rewards. The main effect of error_n-1_ was not significant (*b* = 0.03; *z = *0.83; *p* = 0.406). The main effect of task was not significant (*b* = 0.04; *z = *0.99; *p* = 0.320). The interaction of error_n-1_ and task was significant (*b* = 0.09; *z = *2.24; *p* = 0.025), with an increased probability to switch after errors, compared to accurate responses, when performing the motion task. However, this effect reversed when performing the color task with a decreased probability to switch after errors, compared to correct responses.

The result pattern of this main analysis was further explored. Even though participants were instructed that points would decreased with repetitive task repetitions, it might have been that participants had to learn the specific dynamics of the reward structure first. Thus, an exploratory analysis examined whether participants were more sensitive towards reward differences in the second half of the experiment, compared to the first half of the experiment. Therefore, another factor *experiment half* was computed indicating the first and second half of the experiment. Note that the first eight blocks were considered as the first half, while the second nine blocks were considered as the second half. A logistic mixed model with the fixed effects and random slopes reward difference and experiment half as main effects and their interaction and participants as random intercept revealed a significant main effect for reward difference (*b* = 0.32; *z = *4.73; *p* < 0.001) and a significant interaction between reward difference and experiment half (*b* = 0.01; *z = *486; *p* < 0.001). The pattern of this interaction suggested that participants’ probability to switch to the alternative task increased on higher reward differences on trials in the second experiment half. The main effect of experiment half was not significant (*b* = 0.01; *z = *0.74; *p* = 0.941).

## Discussion

In Experiment 1, we investigated whether participants’ voluntary task choice would be influenced by a dynamically changing reward structure leading to reward differences between the performed and the alternative task. Importantly, the voluntary task switching paradigm with double registration, where participants first select a task and then perform the selected task, allowed us to examine the effects of reward differences independent from task performance, as rewards were provided for task selection, but not for task performance. In addition to reward differences, we investigated the influence of errors in n-1 on voluntary task choices. We found that participants were more likely to switch to an alternative task as the alternative task paid more than the current task. These results are in line with prior research on the influence of reward on voluntary task switches (e.g., Braun & Arrington, 2018). However, previous studies provided participants with performance-contingent rewards. Here, rewards were gained for task selection, but not task performance, allowing us to ask whether task performance additionally influenced voluntary task choices.

Against our expectations, we did not observe any effects of errors on the previous trial on participants’ task selection behavior. However, this may have been due to the overall small occurrences of errors on both tasks throughout the experiment (color task: 9.8% error rate, motion task: 4.6% error rate). We also observed no task effects on voluntary task choices. Yet, we observed a significant interaction effect error_n-1_ and task which we sought to further explore in the following experiments. A further post hoc analysis revealed that the effect of reward difference was facilitated during the second half of the experiment, suggesting that participants were more sensitive to higher reward differences on later trials in the experiment. Finally, we observed performance differences between the two tasks. However, these effects were rather small with 37 ms RT difference and 5% error rate difference between the two tasks. We considered to increase this difference in the following experiment.

## Experiment 2

While both tasks in Experiment 1 differed significantly from each other in terms of RTs and error rates, it may be that this difference was not sufficiently pronounced to influence participants’ task choices (they were initially set to be similar in RTs and error rates). Thus, we conducted a second experiment with a stronger difference in tasks to (a) replicate the results of Experiment 1 of task selection contingent but task performance non-contingent reward differences influencing the probability to switch to the alternative task, and (b) further investigate whether performance costs influence participants’ decision to switch to the alternative task even if task performance was not rewarded.

We made several adjustments in Experiment 2. First, we reduced the signal-to-noise ratio of both tasks yet to a stronger degree for the color task (motion = 25%; color task 55%). With this change, we aimed to (a) increase the error rate of both tasks, to better investigate the influence of recent errors, and (b) increase the difference in error rate between the two tasks. Second, we carried out Experiment 2 as an online experiment in wake of the COVID-19 pandemic. Third, we reduced the number of blocks to 14 blocks as we expected that performing 17 blocks may be too long for an online experiment. Finally, based on the additional analysis suggesting that participants were more sensitive to reward differences in later trials in the experiment, we first exposed participants to seven blocks (50 trials each) with response contingent feedback (correct/incorrect) after the task execution response, followed by seven blocks (50 trials each) without feedback. We only analyzed the final seven blocks on which no feedback was provided.

We expected to replicate results from Experiment 1, i.e., that reward differences influence the choice to switch to the alternative task. In addition, we sought to investigate the effect of error commissions and expected higher probabilities to switch to the alternative task after errors compared to accurate responses. Finally, we asked whether the increased difference in error rates between the tasks (due to the signal-to-noise ratio settings) would bias participants’ voluntary choice to switch to the alternative task in this experiment. Based on prior findings, we expected participants to trade-off monetary performance non-contingent rewards with reward non-contingent performance costs—reflected with participants to switch to the alternative task with lower reward differences when the alternative task was associated with lower error rates (indicating lower cognitive costs) and participants switching to the alternative task with higher reward differences when the alternative task was associated with higher error rates (indicating higher cognitive costs). We also predicted increased switch rates following errors compared to correct responses. These predictions suggested additive effects of the three factors, however, we also investigated the multiplicative nature of these effects by exploring the interactions between them. The present study was not preregistered.

## Method

### Transparency and openness

The raw data and analysis scripts of all experiments are available via the Open Science Framework at https://osf.io/epx8b/. The present study was not preregistered. The data were collected in January 2021. This experiment considered a target population of 18–45-year-old right-handed males and females from Germany.

### Participants

Fifty-four right-handed participants (29 females; mean age = 31.23; SD = 4.58) were from Germany and recruited with Prolific to participate in this online experiment. However, 18 participants did not meet the inclusion criteria and were thus excluded prior to the data analysis. The final sample thus included 36 participants. Participants earned five Euros for the 45 min study and were able to increase this base rate with a task choice-dependent bonus payment summing up to a maximum of 7 Euros in total for this experiment. All participants signed a consent form and agreed on sharing their data at the beginning of the experiment.

The sample size was based on the sample size of Experiment 1. An a priori power analysis based on 100 simulations and data collected in Experiment 1 was calculated with the simr package (Green & Macleod, 2016). It revealed a power of 89% with a 95%-confidence interval (95%-CI) between 85 and 93 for the observed effect for reward difference in Experiment 1 with an effect size (beta) of 0.02 and an alpha level of 0.05. In addition, we simulated an a priori power analysis to estimate the power of a minimal effect for the task effect in this experiment. This power analysis was based on 100 simulations and suggested a power of 83% with a 95%-confidence interval (95%-CI) between 79 and 86% for a minimal effect size of 0.4 and an alpha level of 0.05.

### Stimulus, procedure, variables, and data analysis

The same paradigm as in Experiment 1 was applied with the following minor adjustments. The signal-to-noise ratio of the color task was set to 55% (55% of the dots in the target color and 45% of the dots in the other color), and 25% for the motion task with 25% of the dots moving in the target direction and the remaining in a random direction. As in Experiment 1, participants had 1000 ms to respond. Participants responded to a total of 14 blocks, each of which included 50 trials with self-paced breaks between the blocks. In the first seven blocks, participants were provided with response contingent feedback, while no feedback was provided during the final seven blocks. Only trials on which no feedback was provided were analyzed. The same variables were used, and the same data analysis was conducted as in Experiment 1. As in Experiment 1, the two random slope variables reward difference and task revealed the best model fit. In addition, a random intercept for participants was fitted to account for variability between participants on their overall probability to switch to the alternative task.

## Results

The effects of task on participants’ RT and error rates are plotted in Fig. 3. The results of the main analysis are shown in Fig. 4. Seven participants who responded below an accuracy of 55% and 11 participants who never switched tasks were excluded from the data analysis, summing up to a final sample that included 36 participants. The first trial of each block (2% of the trials), all responses below 200 ms (12.1%) and all trials without a response (7.7%) or a task selection (0.6%) were excluded. The average switch rate was 6.67% (*SD* = 7.13). Also see S1 for an individual difference analysis on how participants’ RT and error rate differences between the two tasks affected their task choice.

### Average task choices, task choice latencies, rts, and error rates

The motion task was selected with an average of 56.98% (SD = 16.41) of the trials. This result suggests that the motion task (associated with relatively low error rates) was selected more often than the color task (associated with relatively high error rates). There was no significant main effect of task on task choice latency (*b* = 4.93; *t* = 0.91; *p* = 0.368). Responses were not significantly faster on the motion task compared to the color task (*b* = -9.73; *t* = -1.13; *p* = 0.265), with an average response time of 611.7 ms for the motion task and 631.2 ms for the color task. Error rates were significantly lower on the motion task compared to the color task (*b* = -0.44; *z = *-5.97; *p* < 0.001), with an average of 13.0% error rate on the motion task and an average of 26.6% error rate on the color task. This error rate difference estimated with a logistic mixed model was significant between experiments (*b* = 0.62; *z = *4.06; *p* < 0.001), revealing an error rates difference of over 13% in Experiment 2 which was substantially larger than in Experiment 1 in which error rates differed by less than 5%. This also suggested that increasing the signal-to-noise ratio difference between both tasks successfully increased the difference in error commission between the two tasks.

### Main analysis

The probability to switch to the alternative task as a function of reward, error_*n *− 1_, and task*.* In line with the results of Experiment 1, the main effect of reward difference was significant (*b* = 0.11; *z = *8.24; *p* < 0.001), with an increasing probability to switch to the alternative task with a higher difference in rewards. The main effect of error_n-1_ was not significant (*b* = 0.01; *z = *0.24; *p* = 0.806). In line with our expectations, the main effect of task was significant (*b* = − 0.48 *z = *− 4.12; *p* < 0.001) with an increased probability to switch away from the color task (which was associated with a higher error rate) compared to the motion task (which was associated with a lower error rate) than vice versa. No other interactions were significant.

Upon reviewers’ request, we also report the results of the full dataset, i.e., including the first half of the experiment where participants received response contingent feedback. The results of this analysis were similar to the results reported in the manuscript, with significant main effects of reward difference (*b* = 0.07; *z = *8.37; *p* < 0.001) and task (*b* = − 0.26; *z = *− 2.13; *p* = 0.032). However, we observed a significant interaction between reward difference and task (*b* = 0.005; *z = *2.13; *p* = 0.009). This interaction showed that participants consider cognitive costs due to error rate difference more on small reward differences, compared to large reward differences, suggesting that rewards and cognitive costs are not purely considered in an additive fashion but also in a multiplicative fashion.

## Discussion

The purpose of Experiment 2 was twofold. First, we sought to replicate the result of the main analysis of Experiment 1 of higher probabilities to switch to the alternative task with increasing reward differences. Second, we asked whether the probability to switch to the alternative task would be influenced beyond monetary rewards in terms of stark differences in error rates between tasks and recent errors. Results of our manipulation check revealed that the task manipulation of Experiment 2 substantially increased the difference in error rates between the two tasks, with a 13% error rate difference between tasks. In addition, participants avoided performing the color task (which was associated with a higher error rate) by selecting the motion task 56% of the time. With regards to our first objective of the main analyses, results replicated the finding of Experiment 1 of increased switch probabilities to the alternative task with increasing reward differences. With regards to the second objective, results suggested that participants’ performance costs associated with the tasks significantly influenced their task choice, with a higher probability to switch away from the color task (associated with a higher error rate) to the motion task (associated with a lower error rate) than vice versa. This comports with recent theories which suggest that task selections are based on a trade-off between performance costs and monetary rewards to select the task with the highest expected value (e.g., Shenhav et al., 2013). Moreover, the significant interaction observed in the analysis of the full dataset point toward a multiplicative computation of costs and benefits when facing voluntary task choices. In particular, the results suggest that on small reward differences, cognitive costs are more taken into account, compared to rewards, than on larger reward differences. However, as in Experiment 1, we did not find evidence for errors in n-1 s to influence voluntary task choices significantly. Finally, the interaction results of reward difference and recent errors observed in Experiment 1 did not replicate in this experiment.

In sum, the results of Experiment 2 provided evidence that the decision to switch to an alternative task was driven by differences in rewards, but also by reward-independent performance costs, in terms of stark differences in error rates between tasks. To assess the robustness of these results, we attempted to replicate them in a third experiment.

## Experiment 3

The purpose of Experiment 3 was to test the robustness of the results obtained in Experiment 2. In addition, we sought to test whether these results would hold by changing the signal-to-noise ratio that the motion task is associated with a higher error rate and the color task is associated with a lower error rate. A replication of the results of Experiment 2 but with a flip in error rate difference between the two tasks could provide further support that error rate differences influenced voluntary task choices and could rule out alternative arguments such as that the identity of the two tasks (color discrimination vs. motion discrimination) influenced voluntary task choices. The present study was not preregistered.

## Method

### Transparency and openness

The raw data and analysis scripts of all experiments are available via the Open Science Framework at https://osf.io/epx8b/. The present study was not preregistered. The data were collected in January 2021. This experiment considered a target population of 18–45-year-old right-handed males and females from Germany.

### Participants

Forty-six right-handed participants (26 females; mean age = 27.56; SD = 5.32) with normal or corrected to normal vision were recruited from Prolific to participate in this online experiment. All participants signed a consent form and agreed on sharing their data at the beginning of the experiment. Nine participants were excluded prior to data analysis based on the same criteria as in the previous experiments. The total number of participants was 37 (similar to the sample size of Experiment 2). Participants earned 5 Euros for the 45 min study and were able to increase this base rate with a task choice-dependent bonus payment summing up to a maximum of 7 Euros in total.

The sample size was based on the sample size of Experiment 2. An a priori power analysis based on 100 simulations and data of Experiment 2 was calculated with the simr package (Green & Macleod, 2016). This simulation revealed a power of 93% with a 95%-confidence interval (95%-CI) between 88 and 97 for the observed effect for reward difference in Experiment 2 with an effect size (beta) of 0.11 and an alpha level of 0.05. We also simulated an a priori power analysis to estimate the power of the task effect observed in Experiment 2. This power analysis was based on 100 simulations and suggested a power of 89% with a 95%-confidence interval (95%-CI) between 83 and 93% for an estimated effect size of 0.48 and an alpha level of 0.05.

### Stimulus, procedure, variables, and data analysis

The same paradigm as in Experiment 2 was applied with the following adjustments. To reverse the error rates for both tasks, the signal-to-noise ratio for the motion task was decreased to 15% of the dots moving in the target direction and the remaining in a random direction to increase the error rate for this task. The signal-to-noise ratio of the color task was set to 65% of the dots in the target color and 35% of the dots in the other color to decrease the error rate for this task. The same variables were used, the same data analysis was conducted as in Experiment 2, and only trials where no feedback was provided were analyzed.

## Results

The effects of task on participants’ task performance (RTs and error rates) are depicted in Fig. 3. The results of the main analysis are depicted in Fig. 4. The same exclusion criteria as in Experiment 2 were applied. All participants responded with an accuracy of over 55%, but four participants never switched between tasks and were thus excluded from the data analysis. In the first trial of each block (2% of the trials), all responses below 200 ms (7.3%) and as well as all trials without a response (5.8%) or a task selection (0.5%) were excluded prior to data analysis. The average switch rate was 8.2% (*SD* = 15.5). Please also see the individual difference analysis reported in S1.

### Average task choices, task choice latencies, RTs, and error rates

The motion task was selected with an average of 40.87% (SD = 12.05) of the trials. This result suggests that the motion task (associated with relatively high error rates) was selected less often than the color task (associated with relatively low error rates). There was a significant main effect of task on task choice latency (*b* = 37.96; *t* = 4.16; *p* < 0.001), with faster times to choose the color task (associated with relatively low error rates) than the motion task (associated with relatively high error rates). The performance difference between both tasks reversed in this experiment compared to the previous two experiments. RTs were significantly slower on the motion task compared to the color task (*b* = 40.73; *t* = 4.30; *p* < 0.001), with an average response time of 635.6 ms for the motion task and 554.2 ms for the color task. Error rates were significantly higher on the motion task compared to the color task (*b* = 1.33; *z = *14.22; *p* < 0.001), with an average of 24.7% error rate on the motion task and an average of 2.2% error rate on the color task. A logistic mixed model comparing the error rates of Experiment 3 with Experiment 2 revealed a significant main effect for experiment (*b* = − 1.29; *z = *15.66; *p* < 0.001), suggesting an increase in error rates in Experiment 3 compared to Experiment 2. In particular, the error rate difference of over 22% in Experiment 3 was substantially larger compared to Experiment 2 which was 13%.

### Main analysis

The probability to switch to the alternative task as a function of reward, error_*n *− 1_, and task*.* The main effect of reward difference was significant (*b* = 0.20; *z = *10.63; *p* < 0.001), with an increasing probability to switch to the alternative task with higher difference in rewards. The main effect of task was significant (*b* = 1.68; *z = *5.01; *p* < 0.001), with a lower probability to switch to the alternative motion task when performing the color (associated with a lower error rate) task than vice versa. The main effect of error_n-1_ was not significant (*b* = 0.04; *z = *0.27; *p* = 0.789). The interaction of reward difference and task was significant (*b* = -0.03; *z = *-3.57; *p* < 0.001). This interaction suggested that participants were less likely to switch to the alternative task with relatively small rewards if the alternative task was associated with higher error rates than the performed task. However, with increasing reward differences, this effect vanished, and participants were similarly likely to switch to the alternative task, on high reward differences, irrespective of the task.

As in Experiment 2, we also analyzed the full dataset, including all trials on which participants received feedback. These results replicated the previous results which included only trials without feedback. A significant main effects of reward difference (*b* = 0.10; *z = *9.22; *p* < 0.001) and task (*b* = 1.33; *z = *9.22; *p* < 0.001) was observed and the interaction between reward and task was significant (Exp 3: *b* = − 0.01; *z = *− 3.86; *p* < 0.001), with participants considering cognitive costs more on low reward differences but less on high reward differences, suggesting that rewards and cognitive costs are not purely considered in an additive fashion but in a multiplicative fashion.

## Discussion

We conducted Experiment 3 to test the robustness of the results of Experiment 2 of a main effect of reward difference and a main effect of task. The results of Experiment 3 successfully replicated the results of Experiment 2 showing higher switch rates to the alternative task with increasing reward differences favoring the alternative task. Moreover, participants were less likely to switch away from the color task (associated with lower error rates) than from the motion task (associated with higher error rates). In addition, we observed an interaction effect that suggested that when participants performed the color task (associated with lower error rates), they were less likely to switch to the motion task (associated with higher error rates) on small reward differences than vice versa. However, on larger reward differences this task effect was reduced and participants revealed similar probabilities to switch to the alternative task, independent of the task. Finally, we found no support for the prediction that errors in n-1 significantly influenced voluntary task choices.

## Between experiment comparison with Experiment 3 of Spitzer et al. (2022)

The first three experiments provided evidence that with changing rewards, switch rates increase. However, as time on task increases with reward differences in our experiment, switch rates might also increase due to boredom effects caused by time on task. Thus, we compared switch rates of each of the first three experiments with switch rates of a control experiment which we recently reported where rewards did not vary (see Spitzer et al., 2022). We expected overall lower switch rates in Experiment 4 compared to each of the first three experiments.

## Method

### Participants

Fifty participants (32 females; Mage = 25.9; SDage = 4.1) participated in this experiment but 13 were excluded, leading to a sample of 37 participants. For a closer description of participants, see Experiment 3 in Spitzer et al. (2022).

### Stimulus, procedure, and data analysis

The stimuli and experimental procedure were similar to each of the three experiments reported above. An 18% motion coherence and a 59% color coherence were applied. These coherences were selected as they led to 20% error rates in previous experiments. Participants were instructed to respond as fast and as accurately as possible. Rewards were not provided for task selection. Participants responded to 10 blocks of 80 trials in this control experiment. Please note that this experiment thus included a few fewer trials (800 in total) compared to Experiment 1 (850 trials) but more trials compared to Experiment 2 and Experiment 3 (700 trials each). However, if boredom or time on task does lead to increased switch rates, then participants should switch more in this control experiment it includes more trials within one block and more trials compared to Experiments 2 and 3.

## Results

**Fig. 5:**
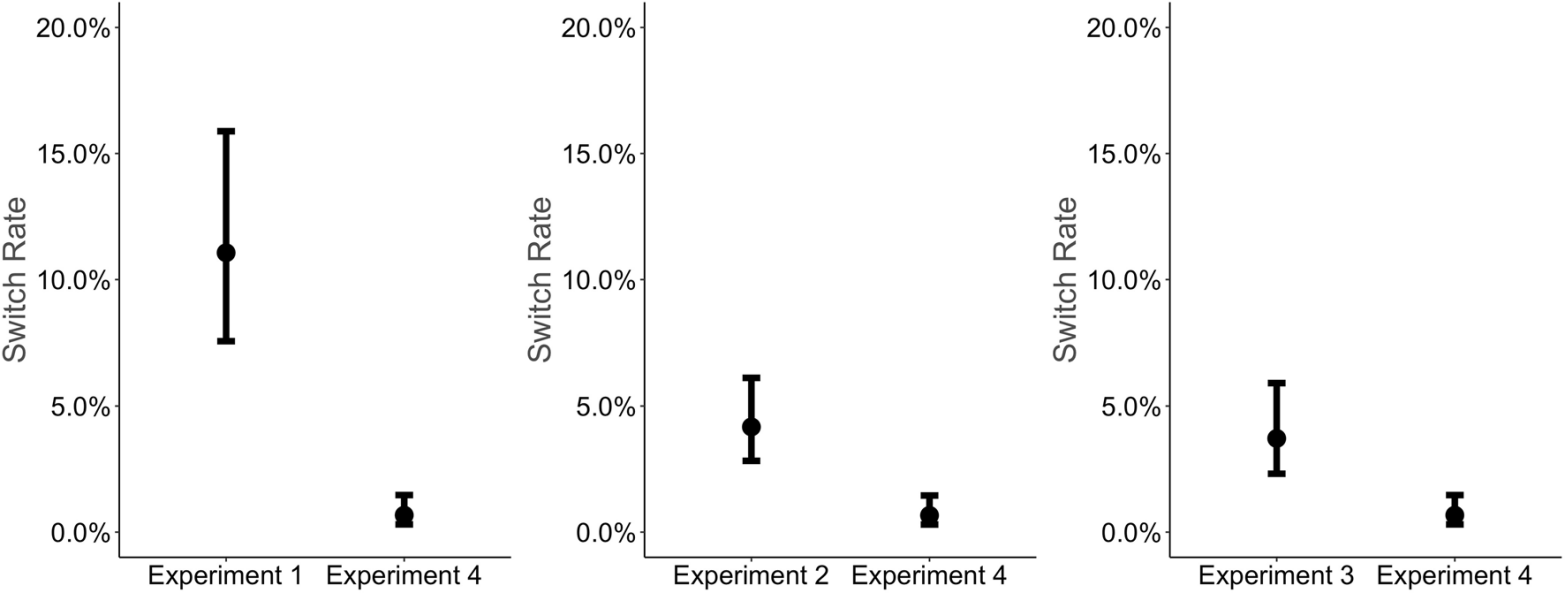
Switch rates as a function of experiment. Switch rates were significantly higher for each experiment with varying rewards compared to a control experiment without varying rewards. Dots depict estimates of the logistic mixed model. Error bars depict the standard error of the mean

Results of the logistic mixed model showed that switch rates were significantly lower in Experiment 4 compared to each of the three other experiments (Exp 1 vs. Exp 4: b = -1.45; *z = *− 6.38; p < 0.001; Exp 2 vs. Exp 4: b = –0.93; *z = *− 4.12; p < 0.001; Exp 3 vs. Exp 4: b = –0.87; *z = *− 3.67; p < 0.001). Together, these results suggest that without reward differences, switch rates were generally lower compared to varying reward differences (see Fig. 5).

## Discussion

The purpose of Experiment 4 was to test whether switch rates were higher in the experiments with varying rewards leading to reward differences between tasks compared to an experiment without varying rewards. Results showed that switch rates of this control experiment were significantly lower compared to each of the previous three experiments with varying reward differences. These results thus rule out that participants get bored with increasing time on task and thus switch to an alternative task. The results rather suggest that without environmental changes, such as varying rewards, participants rarely switch tasks (also see: Arrington & Reiman, 2015; Kessler et al., 2009).

## General discussion

Previous behavioral studies examined the effect of either rewards or errors on decision-making in voluntary task switching. Here, we examined the main effects and interactions between three factors (reward differences, error rates associated with different tasks, and errors in n-1) in a single paradigm. Contrary to prior studies, we implemented a voluntary task switching paradigm in which rewards were solely contingent on task selection, not task performance, to examine the independent influence of reward differences and task performance effects within one experimental paradigm. In other words, we asked whether task performance would still influence participants’ decision to switch to an alternative task even if it does not influence reward optimization. Across three experiments, we found that reward differences influenced voluntary task choices, with higher reward differences favoring the alternative task resulting in a higher probability of switching to the alternative task than the performed task. In Experiments 2 and 3, we also found that large error rate differences between tasks influenced voluntary task choices, with lower error rates yielding higher switch rates to the alternative task, suggesting that task performance influences task choices independent of rewards gained. Our results of Experiments 2 and 3 also showed that this performance difference effect (difference in error rates between the two tasks) cannot be attributed to the identity of the two tasks (color discrimination task vs. motion discrimination task), as the error rates flipped between tasks in Experiment 2 and Experiment 3. The results of Experiment 3 also suggested that the task performance effect on voluntary task choices may be facilitated when the difference in reward associated with the two tasks is small (favoring the alternative task) and may be less severe on larger reward differences (favoring the alternative task). However, this finding was rather exploratory and is worth examining in future work. Finally, we did not observe that errors in n-1 affected voluntary task choices across the three experiments.

In sum, our results provide an empirical basis for future theoretical development on the interplay of the effect of rewards and performance costs on voluntary task choices. This and future empirical work may help inform models' parameterization considering voluntary task choices. We discuss these findings concerning previous work in the remainder of the discussion.

## Performance costs affect voluntary task choices even when rewards are based on task choices, not task performance

Our findings extend previous research showing that error rates associated with a specific task influenced task selection when this task was independent of the rewards, as rewards were gained for task selection and not for task performance. In particular, we expected that reward differences favoring the alternative task should lead to increased switch rates for this alternative task. However, it was unclear if task performance effects, such as error rates associated with a task, and recent errors on the previous trial, would still affect task choices, despite the fact that task performance was detached from task choices. Surprisingly, however, our results showed that task performance still affected participants’ task choices.

A previous behavioral study in which participants were provided with performance-contingent rewards that examined the effect of rewards on task selection excluded erroneous responses (Braun & Arrington, 2018). Our paradigm, however, allows to examine the influence of rewards independent of performance costs on voluntary task choices. The robust finding across three experiments of reward differences influencing participants' voluntary task choices towards the task associated with gaining higher rewards observed in the present study is in line with the findings from Braun and Arrington (2018) and extends these findings by additionally considering the effect of task performance on voluntary reward-dependent task choices.

How rewards and task performance affect task decisions has been investigated in previous research (Chong et al., 2017; Gilzenrat et al., 2010; Westbrook et al., 2013, 2020). The present study extends this literature in several important respects. First, those studies which examined the trial-by-trial effects of rewards and cognitive costs associated with the performance of each task on task decisions varied rewards and cognitive costs *within* the same task and not between tasks (Chong et al., 2017; Gilzenrat et al., 2010; Westbrook et al., 2013, 2020). Therefore, it remained unclear how rewards and task performance inform the decision to switch between different tasks, a situation frequently encountered when deciding between multiple tasks. Second, the present study rewarded participants for task selection independent of task performance. This allowed asking how error rates, as well as errors in n-1, affected voluntary task choices independent of gaining (or missing) rewards. In previous other studies, rewards were performance contingent (Chong et al., 2017; Gilzenrat et al., 2010; Westbrook et al., 2013, 2020). This does not allow for the examination of the independent effect of errors and correct responses on voluntary task choices.^4^ Even though we did not observe any effect of recent errors on voluntary task choices, several recent other studies (not examining rewards in addition to errors) observed an effect of errors in n-1 (for a more detailed discussion on the absence of this effect see below). Third, and finally, the present study showed that rewards motivated participants to switch to the other task, but that additionally, error rates associated with each task were considered in this task choice, with increased error rates on the alternative task biasing participants to forgo rewards and stay longer for a less rewarding but easier task. Moreover, the significant interaction observed in Experiment 2 (all trials) and Experiment 3 points towards the idea that rewards and cognitive costs may be integrated in a multiplicative fashion. Our results provide the first evidence that cognitive costs have a higher impact on task selection when reward differences are low rather than high. This interaction of the effect of rewards and cognitive costs may provide an interesting avenue for future theory development. As such, the present findings extend previous research on within-task decisions substantially, providing evidence that rewards and task performance are considered conjointly when deciding between two tasks.

In Experiment 2 and Experiment 3, error feedback was not provided for the second half of the trials, but participants may have used the error feedback from the first half of the trials to judge the error rate associated with each task. Even though we cannot rule out that participants may have used this explicit feedback from the first half to judge the error rate of each task, a previous study showed that errors affect voluntary task choices even when participants are not given feedback (see Experiment 3: Spitzer et al., 2022). Thus, we assume that the internal error processing in Experiment 2 and Experiment 3 affected participants’ decision to voluntarily switch tasks. The differential effect of explicit error feedback, compared to no error feedback, in combination with the effect of rewards on voluntary task choices may be addressed in future research.

There are several reasons why we may not have observed an effect of errors in *n*−1 on voluntary task choices. Spitzer et al. (2022) investigated the effect of errors in *n* − 1 in combination with increasing error rates and without rewards for task selection and task performance (Spitzer et al., 2022). In contrast, the present study examined the error in *n*−1 effect with two fixed signal-to-noise ratios for each of the two tasks and in combination with varying reward differences. The present results suggest that the effects of reward differences and error rates associated with a task affected voluntary task choices. The effect of these two factors may thus be superseding the potential effect of error commission compared to accurate responses. In addition, Spitzer et al. (2022) only observed n-1 error effects when error rates increased with varying signal-to-noise ratios. Here, error rates were stable across the experiment as the signal-to-noise ratio was kept constant. Thus, it may be the case that the effect of errors in *n*−1 is more likely to be observed when error rates increase—a hypothesis that is worth testing in a reward varying and signal-to-noise varying voluntary task switching paradigm.

Our findings are also in alignment with prior computational (Musslick et al., 2019b) and experimental work (Kool et al., 2010; Spitzer et al., 2022; Westbrook et al., 2013; Wisniewski et al., 2015) suggesting that error rates associated with a task bias voluntary task choices and extend these findings showing that error rate differences between tasks influenced voluntary task choices even when rewards were provided for task choices, but not task performance. Our findings extend previous behavioral findings indicating that performance costs influence voluntary task choices even in situations in which rewards are provided for task selections and not task performance. They also comport with previous models on control allocation suggesting that humans trade-off cognitive costs and rewards associated with each task (Musslick et al., 2015; Shenhav et al., 2013; Silvestrini et al., 2022). These models predict that cognitive agents may switch tasks if the expected value of performing the alternative tasks exceeds that of the currently performed task, such as when the alternative task is associated with a higher reward or if it requires more cognitive control to maintain a high level of performance.

The present results allow a more detailed description of how and when rewards and task performance feed into a shared cost–benefit computation. The observation of independent main effects of reward and errors in all three experiments suggests that agents can use task-related information to attribute costs associated with task performance in a context-specific way. However, the interaction found in Experiment 3 may suggest that under some conditions participants may integrate both reward and error information in a multiplicative fashion, suggesting a more complex decision-making process. Future lines of research would benefit from evaluating the robustness of this interaction to shed more light on the specific integration of costs and benefits into decisions about which task to perform.

The signal-to-noise ratio manipulation in Experiment 2 and Experiment 3 not only affected participants’ error rates but also their RTs (i.e., tasks with increased error rates were also accompanied by increased RTs). In other words, performance costs were not only manipulated between these two experiments in terms of error rates but also in terms of RTs. Participants were paid a fixed amount for their participation and thus, quicker RTs could lead to an increased payrate. As such, participants’ task selection could have also been motivated by an attempt to minimize time on task to finish the experiment as quickly as possible to move on to the next experiment (especially for Experiment 2 and Experiment 3 as these were conducted online). This, in turn, suggests that performing a difficult task not only increased participants’ performance costs (due to relatively high error rates) but also decreased their payrate (due to relatively high RTs). However, these two potential mechanisms are not mutually exclusive and it may well be that participants’ aimed to optimize both performance costs and time on task. Our experiments were not conducted to dissociate between the potential differential effects of error rates and RTs and thus, we cannot rule our which of these two potential mechanisms participants tried to optimize, if not both. Future research may consider to address whether and how participants weigh performance costs and time on task against each other when voluntarily deciding between two (or more) tasks.

## Summary

We investigated the effect of the three factors reward difference, task, and error_*n *− 1_ on voluntary task choices within one novel reward varying and performance non-contingent voluntary task switching paradigm with double registration. We first examined the effect of reward differences and error_*n *− 1_ on voluntary task choices (Experiment 1) and additionally investigated the effect of error rates associated with each task on voluntary task choices (Experiment 2–3). We found that reward differences influenced participants’ voluntary choice to switch to the task associated with gaining higher rewards (Experiments 1–3). We also found that high error rates associated with a task biased participants to consider larger reward differences before switching to an alternative task associated with lower error rates. Our results extend previous behavioral findings showing that task performance error rates affect voluntary task choices in situations in which task selection is rewarded but task performance is not rewarded. Together, the results hint toward a multiplicative effect of rewards and task performance costs on voluntary task choices. These findings are in line with modeling predictions suggesting that humans incorporate monetary rewards of the performed task and alternative task and task performance in their voluntary task choices (Musslick et al., 2015, 2019b; Silvestrini et al., 2022). We hope that these findings help to better understand factors contributing to human decision-making.

### Supplementary Information

Below is the link to the electronic supplementary material.

Supplementary file1 (DOCX 410 kb)

## Data Availability

We have reported all measured variables, conditions, data exclusions, and how we determined our sample sizes. The raw data and analysis scripts of all experiments are available via the Open Science Framework at https://osf.io/epx8b/.

## Footnotes

1 Please note that in the present study rewards for each task were task selection contingent, but task performance non-contingent. That is, rewards were provided for task selection, but not task performance. We then computed the reward difference between each task. This allowed us to examine the effects of task performance independent of reward differences on voluntary task choice.

2 In contrast to Braun and Arrington (2018) and the present study, participants gained rewards randomly (i.e., rewards were not contingent on participants’ task choice) in these studies and reward maximization was not possible in these studies. We therefore do not further elaborate on these studies.

3 Note that these reward differences were contingent on participants task selection (first registration of response), but task performance non-contingent (second registration of a response).

4 Note that another possibility to assess rewards independent from task performance would be to always provide rewards independent of task performance (correct/incorrect) compared to trials where rewards were never provided.
