# Supplementary material for: Task performance errors and rewards affect voluntary task choices
Source: Psychol Res. 2024 Jan 4;88(3):892–909. doi: 10.1007/s00426-023-01908-7 (PMC10965722; doi:10.1007/s00426-023-01908-7)
Supplement: Supplementary file 1 — Supplementary file1 (DOCX 410 kb) [file 426_2023_1908_MOESM1_ESM.docx]

**Individual differences analysis**

We fitted three linear models (one for each experiment) to predict participants’ *task selection difference* (i.e., the number of color task selections – the number of motion task selections) with participants’ *RT difference* between tasks (i.e., RT color task – RT motion task). We fitted three further linear models (again one for each experiment) to predict participants’ *task selection difference* (i.e., the number of color task selections – the number of motion task selections) with participants’ *error rate difference* between tasks (i.e., error rate color task – error rate motion task). We report the results for each of these linear regression models below. The results of the linear regressions are depicted in S1. Capitalized letters correspond to the letter labels of each subplot of S1.

(A) In Experiment 1, the effect of RT difference was statistically significant and negative (beta = -0.74, t(36) = -2.72, p = 0.010)

(B) In Experiment 1, the effect of error rate difference was statistically significant and negative (beta = -145.87, t(36) = -2.04, p = 0.049).

(C) In Experiment 2, the effect of RT difference was statistically non-significant and negative (beta = -0.24, t(34) = -1.87, p = 0.070).

(D) In Experiment 2, the effect of error rate difference was statistically significant and negative (beta = -223.20, t(34) = -2.72, p = 0.010).

(E) In Experiment 3, the effect of RT difference was statistically non-significant and positive (beta < .001, t(35) = 0.03, p = 0.976)

(F) In Experiment 3, the effect of error rate difference was statistically significant and negative (beta = -158.91, t(35) = -2.78, p = 0.009).


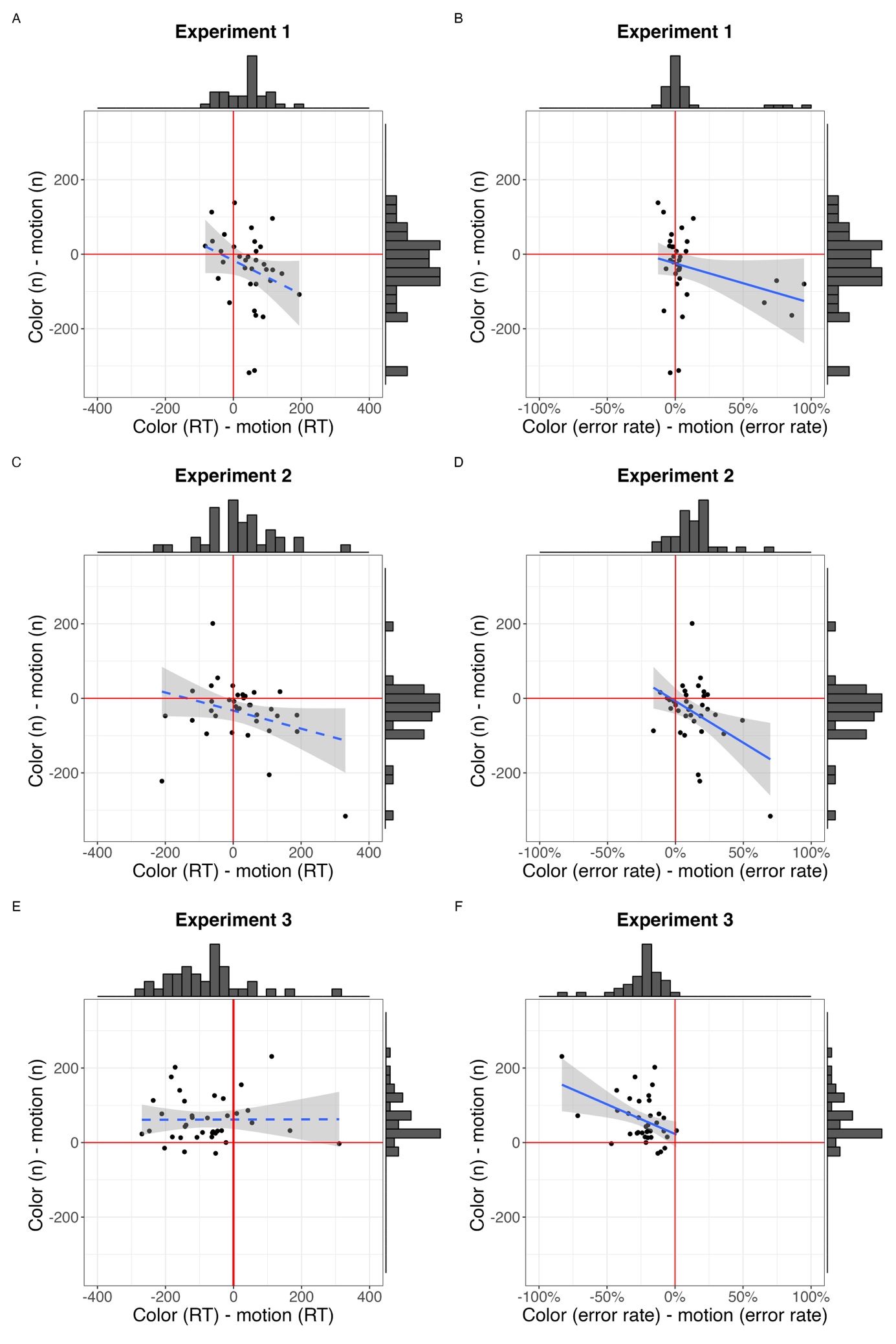


**S1. Individual task differences (dots) in RTs (left panels) and error rates (right panels) and task choice for each experiment.** Each point denotes participants’ average RT or error rate difference (x-axis) between the two tasks and their task selection difference (y-axis). Histograms additionally show the data distribution. Red lines indicate 0 on the x-axis and y-axis. Data points centered around zero in Experiment 1. The motion task was easier in Experiment 2 and thus most of the dots are in the lower right quadrant. This pattern was reversed in Experiment 3; most dots were in the upper left quadrant. We report each of the linear regression results below.
